# Impact of Hypoxia on Relative Biological Effectiveness and Oxygen Enhancement Ratio for a 62-MeV Therapeutic Proton Beam

**DOI:** 10.3390/cancers13122997

**Published:** 2021-06-15

**Authors:** Chun-Chieh Chan, Fang-Hsin Chen, Ya-Yun Hsiao

**Affiliations:** 1 Department of Electrical Engineering, National Chung Hsing University, Taichung 40227, Taiwan; andyccc0915@gmail.com; 2 Department of Medical Imaging and Radiological Sciences, Chang Gung University, Taoyuan 33302, Taiwan; fanghsinchen@mail.cgu.edu.tw; 3 Radiation Biology Research Center, Institute for Radiological Research, Chang Gung University, Taoyuan 33302, Taiwan; 4 Department of Radiation Oncology, Chang Gung Memorial Hospital—Linkou Branch, Taoyuan 33305, Taiwan; 5 Department of Radiology, Chung Shan Medical University Hospital, Taichung 40201, Taiwan; 6 Department of Medical Imaging and Radiological Sciences, Chung Shan Medical University, Taichung 40201, Taiwan

**Keywords:** double strand break, enzymatic double strand break, cell survival, hypoxia, relative biological effectiveness

## Abstract

**Simple Summary:**

This study presents an algorithm to estimate the relative biological effectiveness (RBE) for cell survival under hypoxic conditions using the repair-misrepair-fixation model. The study finds that the RBE values are in the range of 1.0–3.0 for the linear energy transfer equal to 1.1 to 22.6 keV/μm under aerobic condition (21% O_2_) and further increase to the range of 1.1–4.4 under severe hypoxia (0.1% O_2_).

**Abstract:**

This study uses the yields of double-strand breaks (DSBs) to determine the relative biological effectiveness (RBE) of proton beams, using cell survival as a biological endpoint. DSB induction is determined when cells locate at different depths (6 positions) along the track of 62 MeV proton beams. The DNA damage yields are estimated using Monte Carlo Damage Simulation (MCDS) software. The repair outcomes are estimated using Monte Carlo excision repair (MCER) simulations. The RBE for cell survival at different oxygen concentrations is calculated using the repair-misrepair-fixation (RMF) model. Using ^60^Co γ-rays (linear energy transfer (LET) = 2.4 keV/μm) as the reference radiation, the RBE for DSB induction and enzymatic DSB under aerobic condition (21% O_2_) are in the range 1.0–1.5 and 1.0–1.6 along the track depth, respectively. In accord with RBE obtained from experimental data, RMF model-derived RBE values for cell survival are in the range of 1.0–3.0. The oxygen enhancement ratio (OER) for cell survival (10%) decreases from 3.0 to 2.5 as LET increases from 1.1 to 22.6 keV/μm. The RBE values for severe hypoxia (0.1% O_2_) are in the range of 1.1–4.4 as LET increases, indicating greater contributions of direct effects for protons. Compared with photon therapy, the overall effect of 62 MeV proton beams results in greater cell death and is further intensified under hypoxic conditions.

## 1. Introduction

Proton therapy (PT) has been used in radiation therapy (RT) because its dose is specific to the target and surrounding healthy tissues are only slightly affected [1]. The dose is measured using the Bragg curve, whereby the absorbed dose increases gradually and suddenly rises to a peak at the end of the proton track [2]. This feature can be further modulated into a spread-out Bragg peak (SOBP), whereby a uniform dose is delivered at a depth throughout the target volume for clinical RT [3].

An RT treatment plan relies on relative biological effectiveness (RBE) to determine the doses for PT. Currently, the RBE for PT is 1.1, but many studies have demonstrated that this value does not correctly reflect the effectiveness of PT, compared with photon therapy [4,5,6,7]. RBE is closely related to linear energy transfer (LET) [8], and the LET also varies at different positions of the proton track. Several studies show that the LET is largest at the distal edge of the Bragg peak, as is the RBE [5,9,10]. The RBE for protons is 1.1–1.2 at the entrance position and increases to 1.7–3.6 at the Bragg peak when cell survival is taken as the biological endpoint [5,10,11,12]. For other endpoints, such as double-strand break (DSB) induction, the RBE is 1.0–1.1 at the entrance position and mildly increases to 1.2–1.9 at the Bragg peak [9,13]. 

In addition to DSB induction, misrepair of DSBs also affects the total DSB yields. Here, the yields for DSB induction are produced immediately (seconds to minutes) after the time that cells are irradiated by ionizing radiations [8]. Multiple repair pathways are activated when DNA damages are initiated, in which homologous recombination (HR) and non-homologous end-joining (NHEJ) deal with the DSB [14] while base-excision repair (BER) and nucleotide-excision repair (NER) pathways [15,16] deal with non-DSB clustered damage [17]. If those DSBs are not fully repaired, residual DSB can lead to genome instability or cell death [18,19] but do not contribute to additional DSBs. However, extra DSBs could be formatted from the misrepair of non-DSB clustered damage by BER- and NER-associated enzymes and are termed as “enzymatic DSB” [15,20,21]. For example, suppose an unrepaired single-strand break (SSB) is located on a site opposite a damaged base or an apurinic/apyrimidinic (AP) site where the base or AP site is a target for removal in BER pathways. During the removal, the DNA backbone is incised to form a break near an existing SSB, and an enzymatic DSB is then formed. The enzymatic DSB yields for cells irradiated by ^60^Co γ-rays, protons, and helium ions are comparable to the levels of DSB induction; hence it is important to evaluate the yields of enzymatic DSB when RBE is evaluated [22,23,24].

Furthermore, oxygen also plays a vital role in the RBE. DNA damage induction is mainly caused by direct and indirect actions. For low LET radiations, about two-thirds of DNA damage is induced via indirect actions, mostly by hydroxyl radicals (OH) [7]. These radicals R react with oxygen to form RO_2_, which attacks DNA and leads to the formation of DNA damage. The DNA damage is “fixed” and irreversible, which is termed the oxygen fixation hypothesis [8]. The presence of O_2_ modifies the pathway and the final chemical products [25]. Normal cells generally contain higher oxygen concentrations than tumor cells [26]. The level of DNA damage decreases as oxygen concentration decreases [8,27]. Moreover, hypoxia is reported to affect the biology of tumors, including DNA damage induction, repair process, and genomic instability [28,29,30,31].

In RT, mathematical models are able to theoretically predict the RBE for cell survival and provide essential biological information for treatment plans. These models include the local effect model [32,33], the microdosimetric-kinetic model [34,35,36,37], the NanOX model [38,39,40] and the DSB-based repair-misrepair-fixation (RMF) model [41,42,43]. Among these models, the RMF model has been used to predict cell survival for various human cell lines [44] based on the yields for DSB induction and has been applied for treatment planning systems [45,46]. 

Although measured RBE values for cell survival under hypoxia conditions are available [47,48], to our knowledge, we have not found any other simulation to estimate RBE values for PT under hypoxic conditions. This study determines the RBE of a 62 MeV proton beam for DSB induction and cell survival under normal oxygen and hypoxic conditions, respectively, and compares these values with experimental data. The RBE values for cell survival derived by RMF model agree well with the experimental data for AG01522 and U87 cell lines under normal oxygen conditions [10]. Our data also show a significant difference between the RBE values for DSB induction and the values for cell survival, indicating that enzymatic DSB may play an important role in the cause of cell killing.

## 2. Materials and Methods

### 2.1. Energy Spectra

**Figure 1:**
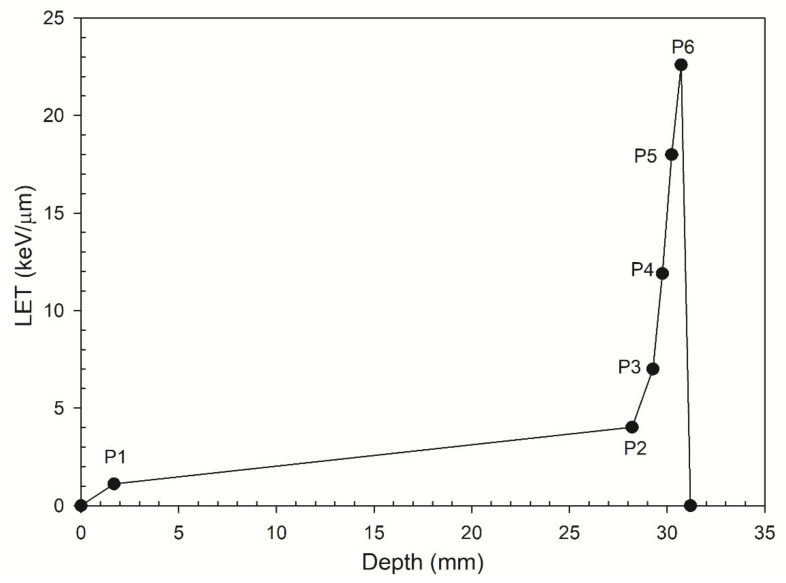
LET versus the depths P1–P6. The depths P1–P6 represent six locations along the track of a 62 MeV proton beam.

All energy spectra for depths P1 to P6 (see Figure 1) are in accord with those from Chaudhary et al. (2014) [10]. They were ungraphed using Image J software, a Java-based image processing software package that was developed at the National Institute of Health [49] using an interval 0.2 MeV.

### 2.2. Monte Carlo Damage Simulation (MCDS)

Monte Carlo simulation is a mathematical technique that applies to stochastic events. It is based on repeated random sampling from numerical simulations, and the results depend on the average outcomes over a large number of runs [50]. The MCDS simulates the yield of DNA damage in a cell irradiated with photons, mono-energetic electrons, protons ions up as heavy as ^56^Fe ions [27,51,52].

MCDS is not a Monte Carlo radiation transport code and cannot simulate the stochastic events generated by ionizing radiations as track structure simulations [53,54,55]. The approach, used in track structure simulations, estimates DNA damage profiles by superimposing DNA geometry to the radiation track structures [55]. This approach is a direct way to estimate DNA damage yields correctly but is more computationally expensive. Alternatively, the other approach is to use the clustering algorithm that MCDS employs. Specifically, MCDS generates random numbers of damage configurations within one cell and simulates the process of DNA damage in two main steps: (1) the initial damage within a cell is randomly distributed in a DNA segment and (2) this damage distribution in a particular segment is subdivided into lesions [51]. The approach used in MCDS is indirectly and reduces the computational time. MCDS estimates the yield for different types of DNA damage and uses reported DNA damage data and the major trends for DNA damage spectra that are similar to those for detailed track structure simulations. Table 1 shows the types and classifications of DNA damage and their abbreviations, including base damage (BD), simple single-strand break (SSB), simple double-strand break (DSB), two or more strand breaks on the same strand (SSB^+^), two or more strand breaks on opposite strands that do not constitute DSB (2SSB), DSBs with additional break(s) on a strand within 10 base pairs (DSB^+^) and more than one DSB within 10 base pairs (DSB^++^).

### 2.3. Monte Carlo Excision Repair Simulation (MCER)

The MCER code simulates the probability of the repair outcomes in the BER and NER pathways for DNA damage in cells that are irradiated with electrons, protons and helium ions [21] and has been applied in several studies [20,23,24]. These pathways include short-patch BER (SP BER), long-patch BER (LP BER), SP BER/NER, and LP BER/NER pathways [20,21]. The repair outcomes were correct repair, repair with a mutation and conversion into a DSB. The third outcome results from the misrepair of some sugars or BD, which convert these non-DSB clusters into DSBs. The details of MCER are described elsewhere [21]. To generate the MCER results, this study used the following parameters for the input conditions: inhibition distance = 8 base pairs; probability of choosing a lesion from the first strand break = 0.5; polymerase error for SPBER = 1.0^−4^; polymerase error for LPBER and NER = 1.0^−6^; probability of incorrect insertion opposite a damaged base = 0.75; probability of incorrect insertion opposite a lost base = 0.75. The values for the parameters mentioned above were suggested by previous studies [20,21].

### 2.4. Calculation of DSB Conversion from DNA Damage

The yield for DSB conversion for proton ions is calculated by the formula below. Enzymatic is defined as the conversion probability for repair pathways for total DNA damage, which is composed of *i* lesions, and *Y_i_* is the yield of the total number of non-DSB clusters per Gy per gigabase pair (per Gy per Gbp), which is composed of *i* lesions, for proton ions with energy *E*.

(1) $$\mathrm{DSB}=\sum_{i}p_{i}(E)Y_{i}(E)$$

### 2.5. RMF Model

An RMF model [41] was developed to link DSB induction to cell survival. The linear-quadratic (LQ) equation [8] based cell survival fraction curve fitting parameters α and β are expressed as:

(2) $$\alpha =\left[1-f_{R}\left(1-\theta \right)\right]\sum +\kappa {\bar{z}}_{F}{\left(f_{R}\sum \right)}^{2}$$

(3) $$\beta =\left(\kappa /2\right){\left(f_{R}\sum \right)}^{2}$$

where $f_{R}$ is the fraction of the initial DSB that are potentially rejoinable and defined below

(4) $$f_{R}=\frac{1}{\sum }\sum_{i=2}^{j-1}\sum_{i}$$

The parameter *j* defines the least number of lesions per DSB that cannot be rejoined, ∑ is the total number of DSB per cell per Gy and $\sum_{i}$ is the expected number of DSB per cell per Gy, which is composed of exactly *i* lesions. The DNA damage yields were converted into the unit per Gy per Gbp using the factor 6 Gbp per cell for a typical mammalian cell [51]. Assuming a continuous slowing down approximation (CSDA), the CSDA range of the particles (62 MeV protons) is above 1.7 cm (calculated by MCDS), which is much greater than the sphere used here (μm). For a uniform irradiation, ${\bar{Z}}_{F}$ is the frequency-mean specific energy for a spherical target composed of water with diameter *d* and is defined as [58]:

(5) $${\bar{Z}}_{F}=0.204\frac{LET}{d^{2}}\left(\frac{keV}{\mu m}\right)$$

The diameter for this study was set as 8 μm. The size of nuclear diameter (~8 μm) was the typical size of V79 Chinese hamster cells used in many experiments [59]. The yields of DSB induction for V79 cells irradiated by ionizing radiations with a wide LET (0.2–520 keV/μm) were used to be the benchmark for MCDS [27].

**Figure 2:**
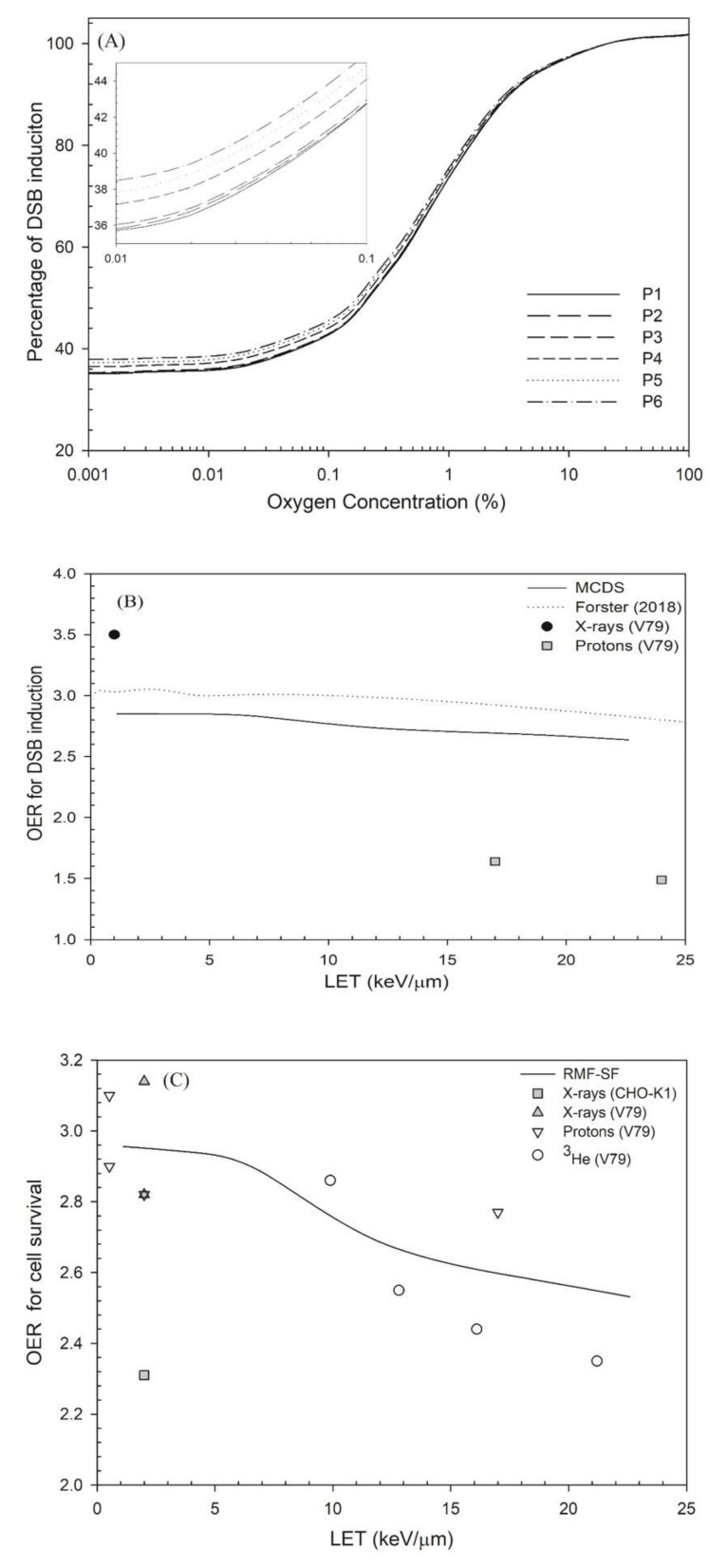
(**a**) Percentage of DSB induction versus oxygen concentration, (**b**) OER for DSB induction using the simulation results of Forster et al. (2018) [60] and this study (MCDS). The experimental data were taken from Prise et al. (1990) [61]. (**c**) OER for cell survival at 10% versus LET. When oxygen concentration decreases to 0.1%, the parameter *j* has a value of 7 to achieve the best fit to the measured OER data. These measured OER values were taken from published studies: CHO-K1 cells [62] and V79 cells irradiated by X-ray [48,61], proton ions [61] and helium ions [63].

θ is defined as the fraction of DSBs that undergo lethal first order misrepair and damage fixation and κ is defined as the fraction of initial DSBs that undergo pairwise damage interactions [41]. For this study, parameter *j* had a value of 9 to achieve the best fit for the measured survival data of AG01522 cells and U87 cells [10] for a LET value of 1−22.6 keV/μm. Parameter *j* = 8 is used in moderate hypoxia (2% O_2_). When oxygen concentration decreased to 0.1%, the parameter *j* had a value of 7 to achieve the best fit to the measured OER data in Figure 2c.

All calculations for RBE for cell survival in a normal oxygen concentration (21% O_2_) or in moderate hypoxia (2% O_2_) [29] used values of θ = 5.79 × 10^−3^ and κ = 5.59 × 10^−5^, which were used in a previous study [41]. For severe hypoxia (0.1% O_2_) [29], the calculations used values of θ = 4.1 × 10^−3^ and κ = 3 × 10^−5^. 

### 2.6. RBE for DSB Induction and Cell Survival

The RBE is defined as the ratio of the dose of low LET reference radiation to the dose of any other radiation that is required to achieve an equal biological effect [8]. The RBE is also expressed as a ratio of the DSB yield, *Σ*, because DSB induction is linearly proportional to the absorbed dose, *D*, up to a hundred Gy under aerobic condition (21% O_2_) [64] and severe hypoxia (0.1% O_2_) [65], as:

(6) $$RBE=\frac{D_{\gamma }}{D_{P}}=\frac{\Sigma_{P}}{\Sigma_{\gamma }}$$

Subscripts *P* and *γ*, respectively, denote protons and γ-rays. The DSB yield for ^60^Co γ-rays is the reference for all reported RBE values.

Cell survival is described using a LQ equation [8]:

(7) $$S=e^{-\left(\alpha D+\beta D^{2}\right)}$$

where *S* denotes the fraction of cells that survive at dose *D* and the curve-fitting parameters are α and β. Using Equation (6) and the RBE definition in Equation (5), the formula for RBE for cell survival [66] becomes:

(8) $$RBE=\frac{\left(\sqrt{\alpha_{\gamma }^{2}+4\beta_{\gamma }D_{P}\left(\alpha_{P}+\beta_{P}D_{P}\right)}-\alpha_{\gamma }\right)}{2\beta_{\gamma }D_{P}}$$

where $\alpha_{\gamma }$, $\beta_{\gamma }$, $\alpha_{P}$, and $\beta_{P}$ are the α and β parameters that are defined in Equations (2) and (3) for ^60^Co γ-rays and proton exposure, respectively, and $D_{P}$ is the dose of protons.

### 2.7. Oxygen Enhancement Ratio (OER)

The effect of oxygen on cells is quantified in terms of the OER, which is the ratio of the hypoxic dose to the aerated dose that is required to achieve the same biological outcome [57]. OER is also defined as the ratio of the biological effect, such as DSB yield or cell deaths, at the same dose [67]. For this study, OER for DSB induction was defined as the ratio of the yield of DSB induction under aerobic conditions (21% O_2_) to the yield under severe hypoxia (0.1% O_2_). To calculate the OER for cell survival (Equation (8)), where the doses, *D_a_* (aerobic condition) and *D_h_* (severe hypoxia) (0.1% O_2_) are calculated using Equations (9) and (10), respectively, as shown below:

(9) $$OER=\frac{D_{h}}{D_{a}}$$

(10) $$D_{a}=\frac{1}{2}\left\{-\left(\frac{\alpha_{a}}{\beta_{a}}\right)+\sqrt{{\left(\frac{\alpha_{a}}{\beta_{a}}\right)}^{2}-\frac{4\left(\alpha_{a}/\beta_{a}\right)\ln\left(S\right)}{\alpha_{a}}}\right\}$$

(11) $$D_{h}=\frac{1}{2}\left\{-\left(\frac{\alpha_{h}}{\beta_{h}}\right)+\sqrt{{\left(\frac{\alpha_{h}}{\beta_{h}}\right)}^{2}-\frac{4\left(\alpha_{h}/\beta_{h}\right)\ln\left(S\right)}{\alpha_{h}}}\right\}$$

where $\alpha_{a}$, $\beta_{a}$, $\alpha_{h}$, and $\beta_{h}$ are the α and β parameters that are defined in Equations (2) and (3), respectively, under aerobic conditions (21% O_2_) and severe hypoxia (0.1% O_2_).

## 3. Results

### 3.1. DSB Induction and Enzymatic DSB Yield at Normal Oxygen Concentrations

Table 2 lists the DNA damage profile for six depths for a mono-energetic 62 MeV proton track.

The limits of the standard deviations for all subtypes of DNA damage (i.e., BD, SSB, SSB^+^, 2SSB, DSB, DSB^+^, and DSB were within 0.2%. As the depth and LET increased, the yield for simpler damage (BD and SSB) decreased whereas the yield for complex damage (SSB^+^, 2SSB, DSB, DSB^+^, and DSB^++^) increased. In total damage, the portion of BD decreased from 68 to 64%, while the portion of simple SSB only slightly increased from 29 to 30%, indicating that the percentage of complex DNA damage increased as the depth increased. That is attributed to the fact that protons are low-LET radiations, which suggests that the damage was distributed spatially sparsely and the majority of the damage was of the simple types such as BD and SSB [68]. However, as LET increased, the energy deposition became denser and localized, and consequently, the yield of base damage reduced and the yields of complex DNA damage such as DSB, DSB^+^, and DSB^++^ increased, as shown in P6 (Bragg peak).

The absolute yields and RBE values for DSB induction predicted by MCDS are shown in Table 3.

The yield for DSB induction for depths P1 to P6 increased as the LET increased but decreased as oxygen concentration decreased. The RBE values for DSB induction at 21% O_2_ were in the range of 1.02–1.52 but increased to 1.02–1.55 at 2% O_2_ and further increased to 1.02–1.62 at 0.1% O_2_. The RBE values for depths P1 to P4 are almost the same under all oxygen concentrations, whereas they increased slightly 7% for positions P5 and P6 (Bragg peak) as oxygen concentration decreased to 0.1%. Furthermore, the RMF model-derived RBE values for cell survival are compared with the experimental data in Table 4.

Table 4 shows that the RBE value for normal AG01522 cell survival for depths P1–P6 was in the range of 1.2–2.7. For radio-resistant U87 cells, the RBE value was in the range of 1.1–3.3. The RMF model predicted the RBE value for cell survival ranging over 1.0–3.0 for depths P1–P6, which was similar to the experimental data. In contrast, the RBE for DSB induction for depths P1–P6 depths was 1.0–1.5, which was much less than the RBE for cell survival.

In Table 5, the probability of correct repair for the LP BER pathway decreases from 0.962 to 0.926 for depths P1 to P6, while the probability of mutation and DSB formation increases from 0.029 to 0.055 and from 0.009 to 0.019, respectively.

Furthermore, repairing efficacy by different repair pathways was studied and all repair outcomes from SP BER, LP BER, SP BER/NER, and LP BER/NER for positions P1 and P6 were listed in Table 6.

In summation, for all repair pathways, the probability of correct repair decreases from 0.983–0.896 (P1) to 0.967–0.818 (P6). Furthermore, the probabilities of mutation and DSB formation increase from 0.008–0.065 (P1) to 0.017–0.110 (P6) and from 0.008–0.038 (P1) to 0.016–0.072 (P6), respectively. The limits of the standard deviations for all probabilities reported in Table 5 and Table 6 are below 0.1%.

Table 7 lists the yields of DNA induction and enzymatic DSB and RBE for DSB induction and enzymatic DSB from LP BER. 

The results show that the yield for DSB induction for depth P1 and P6 are 8.28 per Gbp per Gy to 12.4 per Gbp per Gy, respectively. The yield of the maximum enzymatic DSB for depth P1 and P6 was, respectively, 5.72 per Gbp per Gy and 9.66 per Gbp per Gy, which accounts for 69–78% of the yields for DSB induction. The RBE for DSB induction was in the range of 1.0–1.5 (Table 3), which was similar to the range of 1.0–1.6 for the maximum enzymatic DSB yield (Table 7).

### 3.2. OER and RBE for Cell Survival under Different Oxygen Concentrations

**Figure 3:**
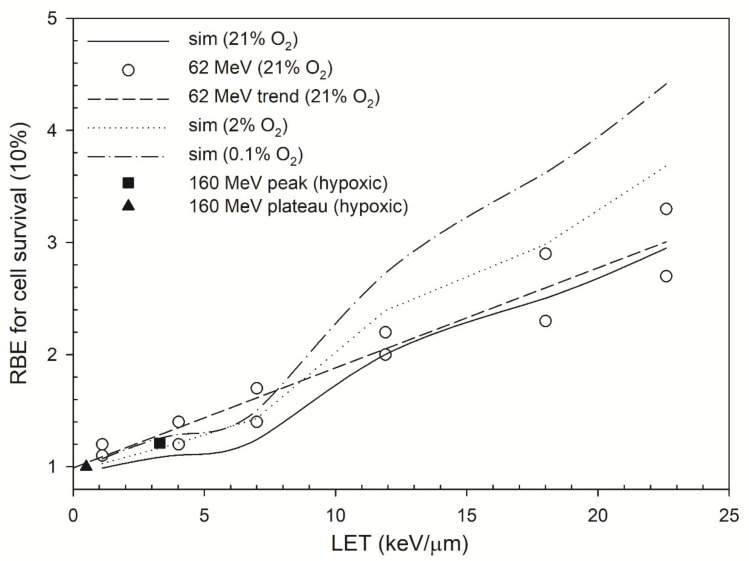
RBE values as a function of LET. The RBE values (dose = 1 Gy) for cell survival at 10% predicted by the RMF model were plotted for oxygen concentrations of 0.1% (dash–dot line), 2% (dotted line) and 21% (solid line). The RBE values were measured under 21% O_2_ (open circle) [10]. This figure also includes the RBE values of 160 MeV proton beams (black square and triangle) [47,48] to represent the RBE values of proton beams under hypoxic conditions (no exact values for oxygen level).

The effects of oxygen on DSB induction and cell survival are shown in Figure 2 and Figure 3, respectively. The yield of DSB induction under severe hypoxia (< 0.1% O_2_) is about 40–45% of the value at normal oxygen concentration (21% O_2_; see Figure 2a). For a 2% oxygen concentration, the yield of DSB induction was about 15% less than the yield at normal oxygen concentrations (21% O_2_). Figure 2b shows that the OER values for DSB induction decreased from 2.85 to 2.6 as LET increases from 1 keV/μm (at P1) to 22.6 keV/μm (at P6) [10], similar to the OER value obtained from Forster’s study [60], 3.0 (1 keV/μm) to 2.9 (22.6 keV/μm). However, the experimental data show that the OER for DSB induction is only 1.5 at LET = 24 keV/μm [61].

Figure 2c shows that the OER for cell survival at 10%under 0.1% O_2_ versus LET. The OER decline for cell survival is more obvious than that for DSB induction. That is, the OER for cell survival decreased from 3.0 to 2.5 as LET increased by 22.6 keV/μm while the OER for DSB induction was in the range of 3.0–2.9. The experimental data showed a decrease from 2.9 to 2.4 as LET increased from 9.9 keV/μm to 21.2 keV/μm [63] for helium ions and a decrease from 2.8 to 2.0 as LET increased from 17 keV/μm to 24 keV/μm for protons [61]. Figure 3 shows that the RBE values for cell survival under normal oxygen conditions were in the range of 1.0–3.0, and the RBE values under 2% oxygen concentration are in the range of 1.0–3.7. Under severe hypoxia (0.1% oxygen concentration), the RBE value for cell survival at depths P1–P6 increased from 1.1 to 4.4.

## 4. Discussion

This study calculates the RBE for DSB induction and cell survival at various oxygen concentrations. The RBE values are compared with experimental data and the results show that the RBE for cell survival is greatly affected by LET (position of the track) and oxygen concentration. The effects of LET and oxygen on RBE are discussed in the following sections.

### 4.1. The Effect of LET on DNA Profile for Depths P1-P6

The MCDS-derived RBE for DSB induction and the RBE value using the RMF model for cell survival has been documented in several studies [37,69], including the comparisons with experimental data for PT [24,37,70]. Compared with the track structure simulations for cells irradiated with lower energy proton beams, such as a 0.3 MeV proton beam (LET = 55.6 keV/μm), the MCDS-derived DNA damage profiles are also similar to those derived by these track structure simulations [51,68,71]. For example, the percentage of BD for 0.3 MeV proton beam by MCDS is 54% [51], which is comparable to the value using track structure simulations, 55% [68]. Recent track structure simulations show that the percentages of DSB, DSB^+^, and DSB^++^ for a 0.3 MeV proton beam are respectively 4.8, 3.6, and 1.3% and total DSB yield is 22.8 per Gbp per Gy [72]. The percentages of DSB, DSB^+^, and DSB^++^ by MCDS (this work) are 4.0, 2.2, and 1.4%, and the total DSB yield is 22.2 per Gbp per Gy, which is comparable to the DNA profiles obtained by track structure simulations. However, other study reported that the measured DSB yields (γ-H2AX foci) are in the range of 5–16 DSBs per Gy per cell (0.8–2.7 per cell per Gbp) for a wide range of LET (1 ~ 270 keV/µm) [73], which is far below than the results obtained by MCDS, 8–25 per Gy per Gbp [41]. This is probably due to that MCDS used the measured DSB yields obtained by pulsed-field gel electrophoresis (PFGE) which tends to have higher yields compared to the number using γ-H2AX methods [73,74].

Table 2 lists the yields of all types of DNA damage in that BD occupies the greatest portion of total damage and is decreasing from 68% (P1) to 64% (P6, with the highest LET), whereas the portions of other types of DNA damage are increasing, although to different levels. Where absolute yield is concerned, both BD and SSB decrease along with the depth of the track while SSB^+^, 2SSB, DSB, DSB^+^, and DSB^++^ increase, indicating that the composition of DNA profiles from P1 to P6 becomes more complex. Other studies using the Geant4-DNA Monte Carlo code also show similar trends for the SSB and DSB yields [75,76]. For example, the SSB yield decreases slowly from 349 per Gy per Gbp to 314 per Gy per Gbp (~9 % reduction) and the DSB yield increases from 13.7 per Gy per Gbp to 22.4 per Gy per Gbp (64% increase) as LET increases to 20 keV/µm [76]. Our results show that the SSB yield decreases from 187 per Gy per Gbp to 170 per Gy per Gbp (~9 % reduction), and the DSB yield increases from 8.3 per Gy per Gbp to 12.4 per Gy per Gbp (49% increase) (Table 2). These findings support the theory that higher-LET radiation produces more complex clustered damage [77]. The yield of complex DNA damage is also related to repair outcomes. When initial DNA damage is clustered and more complex, misrepair or deletion usually occurs because of physical or biochemical interactions, such as oxygen fixation or incomplete repair; then, the RBE for cell survival using the RMF model increases along with the depth (see Equation (2). Therefore, the RBE for DSB induction (in Table 3), as same as the RBE for cell survival, has an increasing trend along with the increase of LET.

### 4.2. Parameter j and α

Under normal oxygen concentration, the only adjustable parameter for the RMF model is parameter *j* (since θ = 5.79 × 10^−3^ and κ = 5.59 × 10^−5^ were determined in a previous study [41]), which defines the least number of lesions per DSB that cannot be rejoined (explained in Materials and Methods). Parameter *j* has been shown to be a constant value of 7 for X-ray data and of 10 for all low-LET radiations (LET <= 20 keV/μm) [41], but the best fit in this study is a value for *j* = 9 for RBE for cell survival of protons at all positions (LET < = 22.6 keV/μm) at normal oxygen concentrations and a value for *j* = 7 at severe hypoxia (0.1% O_2_). These results suggest that a very complex DSB, such as DSB with eight lesions, can be rejoined under normal oxygen concentration. Our results also show that the parameter *j* does not depend on the LET for low-LET radiations (LET < = 22.6 keV/μm), but its value decreases for lower oxygen concentrations (see Figure 2c). For all oxygen concentrations, the fraction of rejoined DSB is in the range of 0.98–0.99, indicating that some DSB (around 2%) cannot be fully rejoined, and it turns out *f*_R_ < 1.

The LQ model parameter *α* has been shown to be linearly proportional to LET by experiments [10]. This relationship can also be derived by the RMF model using the DSB yields by other Monte Carlo simulations [78] (see Appendix A for details). However, as LET increases to certain values, Equation (2) also shows that α does not have a linear relationship with LET since the second term cannot be ignored and α becomes nonlinear [66].

### 4.3. OER

In Figure 2b, the OER value for DSB induction using MCDS is 2.85–2.64 for LET = 1 ~ 22.6 keV/μm, which is in good agreement with the simulated OER value from 3.0 to 2.8 as LET increases to 22.6 keV/μm [60]. Other OER values measured using the filter elution technique are from 3.5 to 1.5 as LET increases from 1 keV/μm to 24 keV/μm [61]. This large difference between the simulation results and experimental measurements may be due to the applied experimental methods in that the result for filter elution-derived OER is somewhat different to the result for PFGE [79]. The filter elution technique was a popular method and has been replaced by newer methods such as PFGE [79]. Most simulations use the DSB yields from PFGE to determine the simulation parameters [80]. In Figure 2c, RMF-model derived OER for cell survival decreases from 3.0 to 2.5 while the measured OER value varies a lot for different radiations. For proton beams, the OER value from the entrance to the SOBP is in the range of 3.0–2.5 (not included in Figure 2c, [81]). For helium ions, the OER value decreases from 2.9 to 2.2 as LET increases from 9.89 keV/μm to 28.5 keV/μm [63], which is in agreement with those obtained by the RMF model.

### 4.4. RBE

Table 3 shows that the RBE value for DSB induction increases from 1.02 to 1.52 as LET increases from 1.11 keV/μm to 22.60 keV/μm and slightly increases to the range of 1.02–1.62 as oxygen decreases from 21% to 0.1%. Other studies also show that the RBE for DSB induction decreases as oxygen concentration decreases [24,27,82]. In Table 4, the RBE for cell survival obtained from the RMF model is in good agreement with the measured RBE for cell survival [10]. These data show that the RBE for DSB induction in severe hypoxia (0.1% O_2_) increases by 7% and the RBE for cell survival using the RMF model increases from 1.0–3.0 to 1.0–3.7as oxygen concentration decreases from 21% to 2% and to 1.1–4.4 as oxygen further decreases to 0.1% (in Figure 3). The relationship between RBE values for DSB induction and cell survival has been discussed previously and indicated that the RBE values for cell survival are generally larger or at least equal to the values for DSB induction [37]. The possible causes for this disagreement suggest that cell killing processes may be involved with other biophysical processes other than DSB induction, such as DNA damage repair, mutation, and genomic aberration [83].

Because the trend of RBE for DSB induction increases as oxygen concentration decreases (Table 3), the increasing trend in the RBE for cell survival under 0.1% O_2_ seems to be correct. However, the RBE values could be overestimated. For example, if the dose required to reach 10% survival for cells irradiated by 62 MeV proton beams in the Bragg peak (P6, measured RBE = 3.3, in Table 4) is 1 Gy (under 21% O_2_), then the dose would increase to 3.3 Gy for cells treated with^60^Co γ-rays Gy (under 21% O_2_) and further increase to 9.9 Gy (measured OER of ^60^Co γ-rays = 3.0, [84]) for cells treated with ^60^Co γ-rays under 0.1% O_2_. The calculated OER value for proton ions (at depth P6) is 2.6 (Figure 2c) and it can be inferred that the RBE (for proton beams relative to ^60^Co γ-rays) under 0.1% O_2_ is around 3.8, which is 16% less than the value, 4.4, derived by the RMF model. These higher RBE values for PT under hypoxic conditions represent the difficulty to efficiently and successfully kill tumor cells by photon therapy since the oxygen level is significantly lower in tumor cells [26].

In Figure 3, the RBE values for cell survival under moderate hypoxia (2% O_2_) are comparable to those under normal oxygen concentration (21% O_2_) and higher RBE values are predicted for cells irradiated at P5 and P6 (Bragg Peak) under severe hypoxia (0.1% O_2_). In contrast, the RBE value for positions P1–P2 (plateau region) only increases slightly. Experimental data also showed that the RBE values of therapeutic proton beams (LET < 5 keV/μm) under hypoxic conditions are about the same (1.0 ~ 1.2) as those under normal oxygen concentration [47,48]. For higher-LET radiations, the RBE for cell survival increases as oxygen concentration decreases, indicating intensified tumor-killing when cells are irradiated under moderate hypoxia (~1% O_2_) [85,86]; this is because the yields of DNA damage induction under hypoxic conditions are mostly obtained by direct actions [24]. The presence of oxygen increases the yields of DNA damage induction via indirect actions, i.e., reacting with free radicals R to form RO_2_. Under hypoxic conditions, the yields of DNA damage induction reduce to one-third of the yields under aerobic conditions for low-LET radiations; however, for higher-LET radiations, the yields of DNA damage induction reduce less because the direct action contributes more in the DNA damage induction. Moreover, the enzymatic DSB yields also reduce due to the reduction in the total damage when oxygen concentration decreases. The overall effects on cells irradiated by proton ions under hypoxic conditions lead to a higher RBE for DSB induction and cell survival.

The study by Lomax et al. (2013) [25] shows that the results of misrepair of DNA damage can be applicable to future RT. Our results show that the yields of enzymatic DSB increase as LET increases (Table 5 and Table 6) and are comparable to the yields of DSB induction (Table 7). It may be possible to target the repair pathway to inhibit the repair of clustered damage in tumor cells by using DNA repair inhibitors such as poly-(ADP-ribose) polymerase (PARP) inhibitors [87,88], leading to an increase in enzymatic DSB yields. In that case, radiosensitizers can be jointly used in the tumor regions to increase the yields in DNA damage induction. For example, we can add oxygen mimetics [89] as a substituent for oxygen in the process of “fixing” DNA damage.

Alternatively, instead of modifying the microenvironment in the tumors, a larger irradiated volume containing a wider SOBP with higher-LET radiations may be applied in RT. This proposal is in line with the idea of “LET-painting” [90], which suggests the redistribution of LET by using different energies of proton beams. In addition, oxygen can be seen as a biomarker to distinguish normal and tumor cells: the median oxygen level in most normal tissues is around 4–7.5%, while the level in tumors is 0.3–4.2% [26]. Because higher-LET proton beams exhibit a higher RBE under hypoxic conditions, the cell killing should be more intensified in tumor regions than that for normal cells. If some radiation scavengers such as dimethylsulfoxide are given into normal cells (located in the outer region of the irradiated volume) to reduce complications, then it is possible to improve the treatment efficiency and reduce complications simultaneously [70].

## 5. Conclusions

This paper presents an algorithm to estimate the RBE for cell survival under hypoxic conditions using the RMF model. This algorithm uses the yields of DSB induction and three parameters (*j*, θ and κ) to calculate the parameters of LQ model, i.e., α and β and further determines the RBE for cell survival. The RBE values determined by this algorithm are comparable to the measured values and could be used in clinical treatment planning for hypoxic tumor cells.

## Figures and Tables

**Table 1:** Description of abbreviations used in this Study.

Full Name	Abbreviation	Description	Reference
Base damage	BD	Isolated base damage	[56]
Simple single-strand break	SSB	Isolated simple strand break	[56]
	SSB^+^	Two or more strand breaks on the same strand	[56]
	2SSB	Two or more strand breaks on opposite strands that do not constitute DSB.	[56]
Simple double-strand break	DSB	Two single-strand breaks on opposite strands but with a separation <10 base pairs	[56]
	DSB^+^	DSBs with additional break(s) on a strand within 10 base pairs (DSB^+^)	[56]
	DSB^++^	More than one DSB within 10 base pairs	[56]
Monte Carlo damage simulation	MCDS	A MC code that simulates the yield of DNA damage in a cellIrradiated with ionizing radiations	[51]
Monte Carlo excision repair simulation	MCER	A MC code that simulates the probability of the repair outcomes in The BER and NER pathways.	[21]
Oxygen enhancement ratio	OER	The ratio of the hypoxic dose to the aerated dose is required to achieve the same biological outcome.	[57]
Relative biological effectiveness	RBE	The ratio of the dose of low LET reference radiation to the dose of any other radiation that is required to achieve an equal biological effect.	[8]
Repair-misrepair-fixation model	RMF model	A mathematical mdoel to link DSB induction to cell survival.	[41]

**Table 2:** Absolute yield of DNA damage (per Gy per Gbp) induced by a 62 MeV proton beam at normal oxygen concentrations (21%).

Absolute Yield (per Gy per Gbp)	BD	SSB	SSB^+^	2SSB	DSB	DSB^+^	DSB^++^	Total SSB	Total DSB	Total Damage
P1	421	178	8.07	1.01	7.19	0.990	0.117	187 ± 0.0246	8.28 ± 0.0109	616 ± 0.0461
P2	405	174	8.80	1.20	7.62	1.15	0.154	184 ± 0.0254	8.93 ± 0.0112	599 ± 0.0506
P3	394	171	9.72	1.48	7.86	1.36	0.213	183 ± 0.0325	9.43 ± 0.0124	585 ± 0.136
P4	367	163	12.3	2.44	8.19	1.94	0.474	178 ± 0.0745	10.6 ± 0.0204	556 ± 0.413
P5	354	159	13.2	2.81	8.44	2.19	0.588	175 ± 0.0870	11.2 ± 0.0229	540 ± 0.475
P6	328	154	13.9	3.08	9.21	2.52	0.668	170 ± 0.0814	12.4 ± 0.0221	511 ± 0.447

**Table 3:** The absolute yield of DNA damage (Gy^−1^Gbp^−1^) and RBE induced by ^60^Co. γ-rays and a 62 MeV proton beam under oxygen concentrations 21, 2, and 0.1%.

Radiation type		21% O_2_		2% O_2_		0.10% O_2_	
Position	LET ^a^ (keV/μm)	DSB (Gy^−1^Gbp^−1^)	RBE	DSB (Gy^−1^Gbp^−1^)	RBE	DSB (Gy^−1^Gbp^−1^)	RBE
P1	1.11	8.28 ± 0.0109	1.02 ± 0.00186	6.97 ± 0.00997	1.02 ± 0.00203	3.54 ± 0.00735	1.02 ± 0.00293
P2	4.02	8.93 ± 0.0112	1.10 ± 0.00182	7.52 ± 0.0103	1.10 ± 0.00199	3.82 ± 0.00765	1.10 ± 0.00288
P3	7.00	9.43 ± 0.0124	1.16 ± 0.00186	7.95 ± 0.0114	1.16 ± 0.00203	4.05 ± 0.00817	1.17 ± 0.00289
P4	11.9	10.6 ± 0.0204	1.30 ± 0.00233	9.00 ± 0.0188	1.31 ± 0.00254	4.68 ± 0.0131	1.35 ± 0.00348
P5	18.0	11.2 ± 0.0229	1.38 ± 0.00243	9.54 ± 0.0214	1.39 ± 0.00267	5.03 ± 0.0152	1.45 ± 0.00366
P6	22.6	12.4 ± 0.0221	1.52 ± 0.00221	10.6 ± 0.0204	1.55 ± 0.00241	5.65 ± 0.0141	1.62 ± 0.00325
^60^Co	2.40 ^b^	8.14 ± 0.0107		6.85 ± 0.00988		3.48 ± 0.00720	

^a^ The values of LET were reported previously by Chaudhary et al. (2014) [10]. ^b^ The value of LET of ^60^Co γ-rays was calculated by a previous study [23].

**Table 4:** RBE for cell survival at 10% Using the RMF model with parameter *j* = 9 and an irradiated dose = 1 Gy under normal oxygen concentration (21% O_2_).

RBE					
Position	LET ^a^ (keV/μm)	DSB Induction (MCDS)	Cell Survival (RMF Model)	Cell Survival AG01522 Cell^a^	Cell Survival U87 Cell^a^
P1	1.11	1.02 ± 0.00186	1.0	1.2	1.1
P2	4.02	1.10 ± 0.00182	1.1	1.4	1.2
P3	7.00	1.16 ± 0.00186	1.2	1.7	1.4
P4	11.90	1.30 ± 0.00233	2.0	2.2	2.0
P5	18.00	1.38 ± 0.00243	2.5	2.3	2.9
P6	22.60	1.52 ± 0.00221	3.0	2.7	3.3
^60^Co	2.40				

^a^ The measured RBE values of AG01522 cells and U87 cells were reported previously by Chaudhary et al. (2014) [10].

**Table 5:** The average repair outcome probability for all types of DNA damage for cells irradiated by a 62 MeV proton beam for LP BER pathway.

Average Repair Outcome Probability for all Types of DNA Damage						
Outcome	P1	P2	P3	P4	P5	P6
Probability of correct repair	0.962	0.958	0.954	0.938	0.932	0.926
Probability of mutation	0.029	0.032	0.035	0.046	0.051	0.055
Probability of DSB formation	0.009	0.010	0.011	0.016	0.017	0.019

**Table 6:** The average repair outcome probability for all types of DNA damage for cells irradiated by a 62 MeV proton beam for SP BER, LP BER, SP BER/NER, and LP BER/NER pathways.

Average Repair Outcome Probability for all Types of DNA Damage						
Outcome	Probability of Correct Repair		Probability of Mutation		Probability of DSB Formation	
Repair Scenario	P1	P6	P1	P6	P1	P6
SP/BER	0.983	0.967	0.008	0.017	0.008	0.016
LP/BER	0.962	0.926	0.029	0.055	0.009	0.019
NER/SP BER	0.899	0.822	0.062	0.106	0.038	0.072
NER/LP BER	0.896	0.818	0.065	0.110	0.038	0.072
Range	0.896–0.983	0.818–0.967	0.008–0.065	0.017–0.110	0.008–0.038	0.016–0.072

**Table 7:** The yields of DNA induction, enzymatic DSB and RBE of DSB induction and enzymatic DSB for LP BER pathway.

	P1	P2	P3	P4	P5	P6
DSB induction (per Gbp per Gy)	8.28	8.93	9.43	10.6	11.2	12.4
Maximum DSB conversion (per Gbp per Gy)	5.72	6.19	6.73	8.77	9.43	9.66
RBE for DSB induction	1.02	1.10	1.16	1.31	1.38	1.53
RBE for DSB conversion ^a^	0.95	1.03	1.12	1.46	1.57	1.61

^a^ The enzymatic DSB yield for ^60^Co γ-rays was reported previously [23], 6.00 Gy^−1^ Gbp^−1^.

## Data Availability

Not applicable.

## Appendix A

The LQ model parameter *α* for cells that are irradiated by proton ions is defined as: $\alpha =\left[1-f_{R}\left(1-\theta \right)\right]\sum +\kappa {\bar{z}}_{F}{\left(f_{R}\sum \right)}^{2}$ (from Equation (2)).

The definitions of all parameters are described in the Materials and Methods Section. Parameter κ is small enough to be ignored ($\kappa =5.59\times 10^{-5}$ and $\theta =5.79\times 10^{-3}$) [41], and $f_{R}\sim 1$ and ${\bar{z}}_{F}\sim 0.01$ so Equation (2) is approximated as:

(A1) $$\alpha =\theta \sum (1+\frac{\kappa }{\theta }*0.01\sum )$$

The value for ∑ (DSB yield) is about 50–100 per cell per Gy, so Equation (A1) is approximated as:

(A2) α=θ∑

DSB induction (per Gbp per Gy) is almost linearly proportional to LET [78]:

(A3) $$\sum =6.8+{\left(0.1835*LET\right)}^{0.9583}$$

the cells are mammalian cells and 1 cell = 6 Gbp (giga basepairs) so the DSB yield is multiplied by 6 and the equation becomes:

(A4) $$\sum =6*6.8+6*{\left(0.1835*LET\right)}^{0.9583}$$

Equation (A2) is rewritten as:

(A5) $$\alpha =\theta (6*6.8)+6*\theta *{\left(0.1835*LET\right)}^{0.9583}$$

This corresponds to the equation, $\alpha_{P}=\alpha_{x}+\lambda *LET$ [10], which is experimentally derived. $\alpha_{P}$ and $\alpha_{x}$ are the respective LQ model parameters *α* for protons and 225 kVp X-rays, and λ is a constant that differs for different cell systems.

Although the power of LET in Equation (A5) is not 1 (0.9583), yet for low-LET radiations, the parameters $\alpha_{x}$ and λ can be respectively approximated to $\alpha_{x}=6*6.8\theta \sim 0.236$ Gy^−1^ and $\lambda \sim 6*\theta *{\left(0.1835\right)}^{0.9583}$ = 0.006842 μm keV^−1^ Gy^−1^. The experimentally derived value for $\alpha_{x}$ is 0.11–0.54 Gy^−1^ while λ is 0.0127–0.0451 μm keV^−1^ Gy^−1^ [10].
